# 

**Published:** 1875-06-01

**Authors:** 


					﻿No. XI.
JUNE 1, 1876.
Vol. XVI.
TIETIE
Midical Examiner:
-A-
Icmi-fflonthlo Journal oil fflfdital fences.
EDITED BY
N. S. DAVIS, M. D., and F. H. DAVIS, M. D.
Subscription Price, Three Dollars per Annum, in advance.
Single Numbers, Fifteen Cents.
CHICAGO.
PUBLISHED AT 65 HL RANDOLPH STREET.
				

